# Beyond Diabetes: Does Obesity-Induced Oxidative Stress Drive the Aging Process?

**DOI:** 10.3390/antiox5030024

**Published:** 2016-07-18

**Authors:** Adam B. Salmon

**Affiliations:** 1 Geriatric Research, Education and Clinical Center, South Texas Veterans Health Care System, San Antonio, TX 78245, USA; salmona@uthscsa.edu; Tel.: +1-210-562-6136; Fax: +1-210-562-6110; 2 The Sam and Ann Barshop Institute for Longevity and Aging Studies, Department of Molecular Medicine, The University of Texas Health Science Center at San Antonio, San Antonio, TX 78245, USA

**Keywords:** reactive oxygen species, mitochondria, inflammation, adipose, fatty acid oxidation, longevity, health span

## Abstract

Despite numerous correlative data, a causative role for oxidative stress in mammalian longevity has remained elusive. However, there is strong evidence that increased oxidative stress is associated with exacerbation of many diseases and pathologies that are also strongly related to advanced age. Obesity, or increased fat accumulation, is one of the most common chronic conditions worldwide and is associated with not only metabolic dysfunction but also increased levels of oxidative stress in vivo. Moreover, obesity is also associated with significantly increased risks of cardiovascular disease, neurological decline and cancer among many other diseases as well as a significantly increased risk of mortality. In this review, we investigate the possible interpretation that the increased incidence of these diseases in obesity may be due to chronic oxidative stress mediating segmental acceleration of the aging process. Understanding how obesity can alter cellular physiology beyond that directly related to metabolic function could open new therapeutic areas of approach to extend the period of healthy aging among people of all body composition.

## 1. Introduction

A large number of diseases, pathologies and other maladies become much more prevalent with advanced age. There is substantial evidence that the molecular regulators of most of these age-related diseases overlap with many of those that are thought to regulate the aging process in general. Now more than ever, understanding how to delay the constant acceleration of aging could profoundly impact health and healthy aging in a growing aged population.

The oxidative stress theory of aging (OSTA) has been exceedingly well-studied as a primary mechanistic theory to describe how the aging process is regulated. Evolution of life in an aerobic environment has afforded species many benefits. However, this lifestyle also increases the likelihood of generating cellular oxidative stress. At the most basic description, cellular oxidative stress is an imbalance between the production of pro-oxidants (including reactive oxygen species (ROS), such as superoxide, hydrogen peroxide, etc.) and the activity of the cellular antioxidant defense (including enzymatic and non-enzymatic antioxidants). More than 60 years ago, Denham Harman developed an original theory suggesting that the oxidative stress associated with aerobic life plays an essential role in the regulation of the aging process [1]. The salient idea of this theory is that chronic levels of oxidative stress lead to oxidative damage to cellular macromolecules that accumulates over time and causes the progressive decline in function of cellular processes that ultimately define aging. Through the years, Dr. Harman continued to develop and refine this theory and identified mitochondria as significant contributors to cellular oxidative stress through their production of superoxide radicals [2] in addition to understanding the importance of multiple different forms of ROS that likely drive oxidative stress in vivo [3]. 

At its most basic, the mechanistic framework of the OSTA predicts (1) that older organisms should exhibit higher levels of oxidative stress and/or damage than younger organisms and (2) that altering levels of oxidative stress should significantly impact the aging process. Thorough literature searches published elsewhere show for the most part that aging is associated with accumulation of oxidative damage to cellular macromolecules, reduced antioxidant defenses and increased susceptibility to oxidative stress in vivo [4,5]. Moreover, these findings have generally held true for multiple organisms tested including standard invertebrate and vertebrate laboratory models, wild-caught organisms and human populations. 

As to the prediction that altering levels of oxidative stress will impact aging, the data from experimental approaches designed to test this prediction are still ambiguous. Studies using genetic manipulation of invertebrate models (largely the roundworm *Caenorhabditis elegans* and the fruit fly *Drosophila melanogaster*) have supported this prediction [6,7], refuted this prediction [8,9,10], and in some cases suggested the opposite of this prediction, i.e., that increasing oxidative stress can extend lifespan [11,12,13]. In mice, the general thrust of data derived from longevity studies regarding genetic mutation of major enzymatic antioxidants has suggested negligible effects on aging (reviewed in [14,15,16]). With few exceptions (discussed further below), altering the expression of these antioxidants in mice does not affect longevity despite mild to dramatic effects on the accumulation of oxidative damage. As we have discussed elsewhere however, modulation of these antioxidant enzymes may have discrete effects on the etiology of different age-related diseases, such as cancer, cardiovascular diseases, metabolic disease, etc., which may or may not be easily dissociated from the lack of effects on longevity [14,15]. The OSTA began as a simple, elegant mechanism describing how aging is regulated; however, the scientific field has discovered this to be far too elementary to adequately describe the complexity of the aging process.

Interestingly, one antioxidant mutant mouse model that has shown repeatable, robust effects on longevity is a mouse generated with complete ablation of superoxide dismutase 1 (Sod1). Several groups have reported that *Sod1*^−/−^ mice have a shorter lifespan (~30%) than control mice, thereby strongly supporting the OSTA [17,18,19]. In most cases, the shorter lifespan of *Sod1*^−/−^ mice may be directly attributed to an increase in cancer rate, particularly of a rare (for C57BL/6 mice) cancer, hepatocellular carcinoma [17,18,19]. However, the appearance of multiple age-related pathologies relatively early in life in *Sod1*^−/−^ mice potentially hint at an acceleration of the aging process as a whole in these mice. These mice have been reported to exhibit acceleration in the rate of development (or increase in the incidence of) multiple pathological changes associated with age-related diseases including sarcopenia and neuromuscular junction degeneration [20,21,22], and pathologies dealing with the ear [23], eye [24,25], pancreatic β-cells [26] and neurons [27]. Why then should the lack of Sod1 drive acceleration of aging, whereas altering the expression of nearly all other antioxidants in mice has no little to no effect on lifespan? One potential reasoning behind this difference is that *Sod1*^−/−^ mice, more so than all other antioxidant mutant mice tested, show a dramatic increase in oxidative damage to cellular macromolecules [18,28,29,30]. For example, oxidation of nucleic acids (as measured by 8-oxo-2’-deoxyguanosine levels in the liver) are 2–3 times higher in aging *Sod1*^−/−^ than in mice lacking glutathione peroxide 1 (Gpx1) or with reductions in other antioxidants such as Sod2 or Gpx4 [15]. Similar increases in lipid and protein oxidation have also been noted [18,28,30]. Thus, it is possible that *Sod1*^−/−^ mice reach a “theoretical threshold limit of oxidative damage” required to accelerate the aging process as a whole, or perhaps to drive the increase in cancer rate, that is unmet in other antioxidant mutant mouse models. 

Digging deeper into other antioxidant mutant mouse models more generally, there is sufficient evidence to suggest that altering the accumulation of oxidative stress/damage does indeed often correlate with changes in health associated with age if not longevity itself [14,15]. To generally sum up these findings, mice with reduction of antioxidant enzymes are usually associated with an increase in incidence, or acceleration of onset, of age-related diseases. Conversely, mice with elevated expression of antioxidant enzymes usually show slowed acceleration or even prevention of many of these diseases even in the absence of changes in lifespan. These seemingly incompatible findings may partly be explained by the particular model organisms used for testing and partly by particular definitions of aging. In regards to the former, laboratory mice are an outstanding resource for many biological studies; however, most strains of mice die almost completely due to neoplasia or neoplasia-related pathologies [31]. Thus, it can be challenging to delineate whether a lack of effect on lifespan is due to a lack of effect on the aging process as a whole or simply a lack of effect on altering the progression of cancer. It is important to reiterate at this point that oxidative stress is known to be critically important to many pathologies, including those that drive various forms of neoplasia, neurodegeneration, cardiovascular disease, liver disease, kidney dysfunction, metabolic diseases including insulin resistance and diabetes, among others [32,33,34,35,36]. While newer evidence suggests that these relationships are by no means unambiguous [37,38], in human populations there is an association between lower levels of oxidative stress and improved health in aging [36,39,40,41]. Thus, it seems likely that oxidative stress plays a role in the regulation of healthy aging if not longevity per se, and both lifespan and health span (including both functional and pathological assessment) likely need quantification for a complete assessment of what we characterize as aging [42]. 

Because natural animal aging does not take place in a vacuum, it is important to also consider the role that external inputs play on the development of age-related disease, if not aging itself. Life on Earth is constantly exposed to numerous generators of ROS both natural (i.e., ultraviolet light, etc.) and man-made (i.e., pesticides, electromagnetic fields, etc.) in addition to the oxidative stress generated by mitochondrial oxidative phosphorylation. Aging experiments in the lab have rightfully focused on elimination of hard-to-control external variables, but it would be remiss to completely dismiss the role these play in healthy aging. In particular, external sources of stress might significantly alter the outcome of longevity experiments through oxidative-stress mediated mechanisms. A potential example of this is in the study of the role of the protein oxidation repair enzyme methionine sulfoxide reductase A (*MsrA*^−/−^) in mammalian longevity. Moskovitz et al. first reported that *MsrA*^−/−^ mice were short-lived; however, even control mice in this study were relatively short-lived for the strain of mice used (a mixed background of C57BL/6 and 129) [43]. One possibility for the short lifespan of control mice was an uncontrolled variable or source of stress in the vivarium that may have limited mouse lifespan. Moreover, it seemed likely that this was ROS-mediated as the lack of MsrA caused a further shortening of lifespan. To address this idea, we repeated the assessment of longevity in *MsrA*^−/−^ mice under conditions designed to maximize control mouse lifespan (i.e., minimize external stress). Under these conditions, we found no effect on longevity in mice lacking MsrA, providing support for the hypothesis described above [44]. Identification of the potential variable is likely impossible, but these studies raise the possibility that some form of controlled stress maintained either for the duration or at specified times in an aging study might yield informative results regarding how we age “normally”. That is, if we could control the source of stress sufficiently, this could be used to determine (1) if these external variables really affect the aging rate or only the development of diseases that limit lifespan and (2) what role oxidative stress plays in the process. Rather than trying to identify vague sources of stress that may exist between animal vivaria, or between the laboratory and the real world, we propose inducing low-level, chronic stress by promoting obesity and metabolic dysfunction using high fat diets for reasons described below.

## 2. Obesity and Oxidative Stress

It is becoming increasingly clear that obesity has become one of the 21st century’s most significant public health concerns. The proportion of those characterized as obese has risen dramatically over the last few decades world-wide, with Western society currently suffering to the greatest degree [45,46]. Much of this is due to sedentary behavior and the availability of high calorie food choices starting even at young ages meaning that many of those suffering from obesity will do so for a significant proportion of their lives. The presence of obesity or excessive fat accumulation is clearly associated with dramatic increases in metabolic diseases and is the primary risk factor for the development of diabetes. However, obesity not only alters the risk of metabolic diseases, but also is associated with increased occurrence and other chronic diseases affecting kidney, the cardiovascular system and cancer [47,48,49]. Not surprisingly then, obesity has been reported to be associated with increased mortality likely due to the elevated incidences of these life-threatening diseases [50], though not equivocally depending on the population [51].

Understanding how obesity alters cellular homeostasis in ways that drive the etiology of these physiological defects could profoundly affect how diseases are prevented and could have significant impact on public health. In this context, there are myriad potential molecular pathways that are affected by obesity in both time- and tissue-specific patterns. One approach to begin to simplify this complexity may be to focus on those common pathways that are (a) affected by obesity and (b) associated with many, or all, the common pathologies associated with obesity. In this regard, we and others have noted that oxidative stress may be a likely target on which to focus. Obesity is known to promote oxidative stress [26,36,40,52,53,54], though it is still unclear whether this is due to the independent pathological condition of obesity or through related alterations to physiological homeostasis such as hyperglycemia and hyperlipidemia. 

Oxidative stress associated with obesity originates from many sources; here we briefly discuss the roles of mitochondrial dysfunction and increased inflammation. Mitochondrial respiration, through the oxidation of glucose and/or fatty acids, can generate oxidative stress through the byproduct of generation of ATP in an oxygenated environment [55,56]. This process can be exacerbated in obesity due to the higher levels of available mitochondrial substrates, i.e., high glucose and fatty acids. The increase of substrates available have been suggested to cause electron back-up during the process of electron transport which, particularly at complex III, can cause superoxide production in and of itself [57]. In line with this basic biochemistry, obesity has been reported to cause mitochondrial dysfunction in multiple forms including elevated levels of reactive oxygen species production [58,59,60,61,62,63,64]. Along with altering mitochondrial function, obesity and accumulating fat mass can cause a pro-oxidative environment by stimulating chronic inflammation. Inflammation generates high levels of free radical production by the immune cells as part of the immune response. Moreover, oxidative stress associated with chronic inflammation and other sources may also promote further production of pro-inflammatory cytokines in a “vicious cycle”. Obesity alters T-cell subsets, particularly in the adipose tissue, and with obesogenic conditions there is a promotion of infiltration and activation of macrophages in the growing adipose tissue [65,66,67,68]. As proof of principle, it has been reported that genetic ablation or pharmaceutical reduction of some of the sources of this chronic inflammation can prevent the incidence of metabolic dysfunction caused by obesity [69,70,71,72]. Presumably, at least some of this beneficial effect could be attributed to the reduction in oxidative stress accompanied with reducing inflammation [73]. 

Taken together, it seems clear that persistence of obesity is associated with chronically increased levels of oxidative stress. Due to the growing numbers of juveniles now classified as clinically obese, there is a growing portion of the population that will remain obese nearly their entire lives and who are thus likely to be show increased risk of developing what we generally term age-related diseases at earlier points in life and reduced health span if not lifespan. It then seems entirely possible that this oxidative stress may be the molecular tipping point in the progression of poor health associated with obesity. Importantly, clarification of oxidative stress at this key role then identifies a potential therapeutic target to slow or prevent the associated health problems in this population group.

## 3. Obesity and Decline in Health Span

The most well-known physiological defect associated with obesity is the development of metabolic disorders, including and primarily type 2 diabetes mellitus (T2DM). The primary etiology in T2DM is the development of insulin resistance, or the pathological decline in insulin response in peripheral tissues [74]. A deficient cellular response to insulin is thought to be the primary deficit associated with obesity and fat accumulation with the main tissues of significance in glucose metabolism being skeletal muscle, liver and adipose tissue [75,76]. While the association between obesity and metabolic dysfunction is well known, the means by which this process is controlled are still unclear. 

Based on observational as well as experimental findings, there is now reasonable evidence that oxidative stress likely plays a key role in the development of insulin resistance and metabolic dysfunction. Perhaps most well-publicized are results from the Framingham Heart Study; several reports from this longitudinal study have identified increased oxidative stress as a significant correlate of metabolic disease [36,39,40,77]. Others too have found significant oxidative damage significantly elevated in those with T2DM [78,79,80]. Importantly, there is also evidence that levels of oxidative damage may predict metabolic dysfunction particularly among the obese, suggesting that oxidative stress is increased prior to the development of insulin resistance [40,80,81]. As described above, obesity is associated with a cellular environment relatively high in oxidant load. We have previously shown that in vivo oxidative damage is significantly elevated in high fat fed, obese mice to a degree roughly equivalent to the increase in oxidative damage associated with old age [15]. The sources of oxidative stress discussed above contribute at least partially, but likely not wholly to the increased levels of oxidative damage. Metabolic dysfunction is also associated with alterations in antioxidant defenses, including both non-enzymatic such as glutathione as well as enzymatic such as superoxide dismutase and glutathione peroxidase [82,83,84,85,86,87]. At least part of this modification of antioxidant defense might be attribute to the negative effects of hyperglycemia on some enzymatic function [88,89]. 

Together, these studies paint a picture in which obesity causes high levels of oxidative stress, though do not necessarily define oxidative stress/damage as the root cause of associated metabolic dysfunction. However, several mouse studies using either genetic or pharmacological interventions have fairly clearly defined the modulation of oxidative stress as a key step between high fat feeding/obesity and the development of metabolic dysfunction. As an example, transgenic mice with an increase in cytosolic (Sod1) or mitochondrial (Sod2) superoxide dismutase, those with an increase in catalase targeted to the mitochondria (mCAT), an increase in peroxiredoxin (Prdx3), those with an increase in cytosolic thioredoxin (Trx1), and those with an increase in mitochondria-targeted methionine sulfoxide reductase A (TgMito MsrA) have all been shown to be protected against the development of insulin resistance when fed high fat diets [53,58,73,90,91,92]. Similarly, treatment with pharmaceuticals that either have antioxidant properties or induce antioxidant expression have been shown to prevent obesity-induced insulin resistance in vivo and oxidant-induced insulin resistance in cell culture models [58,90,93,94]. 

The precise mechanisms by which oxidative stress gives rise to insulin resistance or metabolic dysfunction, though there are likely roles for the common agents of action including induction of stress signaling pathways such as JNK or stimulation of pro-inflammation [95,96,97]. However, there is growing evidence that the effects of oxidative damage to the cell may have an autonomous role on the development of insulin resistance. For example, oxidation of the insulin receptor can diminish the function of this protein in vitro, thereby reducing its ability to respond to insulin signals [98]. Our own studies expanded on this idea using a mouse with a deletion in a key protein oxidation repair enzyme MsrA. Under high fat-fed conditions, we showed that *MsrA*^−/−^ mice became more insulin resistant than expected based on their diet-matched controls [99]. Further, we found that high fat feeding increases the levels of oxidative damage that occur to the insulin receptor in vivo, and that the lack of MsrA exacerbated this damage thereby further reducing insulin receptor function and promoting insulin resistance. Boden et al. have recently defined a similar mechanism in high calorie-fed humans; subjects treated with the high calorie diet showed a rapid onset of insulin resistance that correlated with extensive oxidation of many proteins, including insulin-responsive cellular glucose transporter GLUT4, in adipose tissue [100]. Together, these studies define a crucial, and potentially targetable, role of oxidative stress in the link between obesity and subsequent metabolic dysfunction. 

In addition to diabetes and metabolic dysfunction, there is now ample evidence that obesity can accelerate the progression of other age-related diseases and pathologies in both mouse models of disease as well as normally aging mice [35,36,101,102,103,104,105,106]. For example, several reports have shown high fat diets, or metabolic dysfunction, can accelerate learning and memory loss as well as neurodegeneration in different mouse models of Alzheimer’s disease [107,108,109,110,111]. Moreover, chronic high fat feeding has been shown to increase oxidative stress in the brain and cause cognitive impairment in even normal aged rodents [106,112]. Similarly, many reports have shown an association between high fat feeding and acceleration of multiple forms of cancer in rodent models; moreover, some of these reports have found that reducing obesity-induced oxidative stress can reduce the prevalence or severity of these forms of cancer [113,114]. Similar cases could be made for a variety of other disease states and pathologies including atherosclerosis, kidney disease and cardiovascular disease [115,116,117,118]. 

These findings regarding obesity and disease raise an important, and rather unexplored, question of whether obesity may actually be accelerating the aging process. Paraphrasing the definition of aging established by Miller, aging is a process that converts healthy, young adults into frail, older adults with diminished capacity to resist and increased vulnerability to most diseases and death [119]. As described above, obesity is associated with increased incidence (susceptibility) to myriad diseases and pathologies that would also generally be associated with age-related diseases. In addition, there is ample evidence that obesity, high fat-feeding, or excess fat accumulation is associated with increased mortality in both laboratory and clinical population [48,50,54,120,121,122]. Thus, it is tempting to propose that obesity indeed accelerates the natural process of aging in rodents and humans. On the other hand, a more cautious interpretation might be that the increased prevalence of diseases associated with obesity causes increased likelihood to die because of those diseases but does not fundamentally alter the aging process. There is a fine distinction between these two interpretations; the former suggests all processes associated with aging are accelerated, whereas the latter only connects disease incidence with mortality without concurrently affecting all cellular processes. 

With a relative dearth of data to address this conflict, we recently published a study designed to assess the effects of high fat diet-induced obesity on the health span of mice using a broad approach [54]. In this study, we introduced adult (8 mo.) C57BL/6 mice to a diet containing 45% fat and relatively high levels of carbohydrates (in comparison to standard chow) to induce obesity for the remainder of their life. In some ways, this is similar to what happens among many adults in Western civilization; that is, being of normal weight early in life and at some point in early adulthood a steady increase in body weight and adiposity due to several factors. As predicted from numerous studies, this high fat/high sugar diet induced rapid weight gain in C57BL/6 mice due primarily to excess accumulation of adipose mass. Importantly, and consistent with the idea that obesity causes oxidative stress, we also found increased levels of oxidative stress in vivo. High fat fed mice had significantly higher levels of lipid oxidation (F_2_-isoprostanes) and protein oxidation (protein-bound 4-hydroxynonenal) and suggestion of elevated levels of circulating pro-inflammatory cytokines. These high levels of oxidative stress were associated with a significant increase in glucose intolerance and increased plasma insulin concentrations, both indicative of metabolic dysfunction consistent with insulin resistance. Most relevant to this review, we also found a significant lifespan shortening of approximately 25% in mice fed the high fat diet. This lifespan effect was in similar size and direction to previous studies comparing lifespan of rodents fed various high fat diets compared to normal diet-fed controls [120,121]. 

During the course of this study, we assessed the performance of these mice in tests of several physiological parameters in a longitudinal fashion, i.e., after 4 months on high fat diet (or 12 months of age) and again after 10 months on high fat diet (or 18 months of age, or roughly mid-age for a C57BL/6 mouse). We selected a serious of physiological tests that were, prior to this study, not thought to be largely related to the increased fat accumulation such as the relationship between insulin resistance and obesity. Thus, we designed this study to address the effect of aging, diet and their interaction to whether obesity, and the associated chronic oxidative stress and inflammation, altered the age-related trajectories of physiological performance. For example, after 10 months of feeding, high fat-fed mice showed a significant decline in activity whereas control-fed mice activity was unchanged. Similarly, the swing-time of both front and rear limbs measured during gait analysis was significantly reduced by high fat feeding over this time period and unchanged in control fed mice. In addition, mice fed a high fat diet for 10 months showed a significantly reduced ability to stay on a rotating rod, a marker of balance and coordination. We found the significance of these differences to exist even after accounting for differences in body weight and fat mass. Further, these markers have been reported to decline with normal aging in mice; thus, obesity caused by a high fat diet accelerated this decline in function and contributed to a decline in health span. The exact nature of these declines are unclear, i.e., are they due to dysfunctional muscles, or motor neurons, or other, and this is one of the significant challenges in future studies. In addition, ongoing studies are addressing whether these declines in function can be attributed directly to the increased levels of oxidative stress/damage. The larger question remains though whether this increased incidence of disease is evidence for acceleration of the aging process, if not wholly then at least segmentally? While still unclear, the prevalence of data certainly suggests this and, moreover, implicates oxidative stress as a key player in this process. 

## 4. Conclusions

Ultimately, the goal is to improve the period of healthy aging in all people which will include both the lean and metabolically normal as well as the obese and metabolically compromised. The data above point to the idea that perhaps addressing oxidative stress, either by preventing it through dietary interventions or reducing it through pharmaceutical antioxidants, may be a potential therapeutic approach in this process. On the other hand, mouse studies have largely shown no effect of altering oxidative stress on longevity in lean, healthy rodents and antioxidant treatment has largely failed as a human intervention against obesity and metabolic dysfunction [15,123]. However, as discussed above, perhaps the lack of significant findings in these studies has been limited by their designed approaches. For example, would the lifespan of high fat-fed, obese mice be extended by reducing oxidative stress? Or could antioxidant treatment be used in obese patients with metabolic dysfunction to prevent the progression of additional co-morbidities or slow the acceleration of the aging process caused by chronic inflammation. With the low compliance rate for dietary or exercise changes in this group of patients, identifying potential therapeutics that both improve glucose metabolism as well as slow the progression of associated pathologies is an important target. Perhaps re-exploring a now 60+ year theory on why we age may be viable means to develop some of these therapeutic options.
